# Chronic exposure of Hawaii Island spinner dolphins (*Stenella longirostris*) to human activities

**DOI:** 10.1098/rsos.171506

**Published:** 2018-10-24

**Authors:** Julian A. Tyne, Fredrik Christiansen, Heather L. Heenehan, David W. Johnston, Lars Bejder

**Affiliations:** 1 Aquatic Megafauna Research Unit, School of Veterinary and Life Sciences, Murdoch University, 90 South Street, Murdoch, Western Australia, Australia; 2 Division of Marine Science and Conservation, Nicholas School of the Environment, Duke University Marine Lab, 135 Duke Marine Lab Road, Beaufort, NC, USA; 3 Marine Mammal Research Program, Hawaii Institute of Marine Biology, University of Hawaii, Hawaii, HI, USA

**Keywords:** cumulative exposure, cumulative activity budget, conservation and management, tourism impacts, behavioural disturbance

## Abstract

Habitat selection is strongly influenced by spatial variations in habitat quality and predation risk. Repeated exposure of wildlife to anthropogenic activities in important habitats may affect habitat selection, leading to negative biological consequences. We quantified the cumulative human exposure of a small, genetically isolated and behaviourally constrained spinner dolphin (*Stenella longirostris*) population, off Hawaii Island, and exposure effects on their daytime cumulative activity budget. Dolphins were exposed to human activities within 100 m for 82.7% of the daytime, with a median duration of 10 min between exposure events. Individual dolphins spent on average 61.7% (s.d. = 6.5) of their daytime resting. Of their total rest time, greater than 90% occurred inside sheltered bays. Despite high levels of human exposure, we did not observe an effect on dolphin resting behaviour. The short intervals between exposure events probably prevent dolphins from returning to a natural resting state before the next event. Consequently, ‘control’ observations may represent a resting behaviour of a more vigilant nature. Chronic levels of exposure to human activities could lead to rest deprivation, displacement from preferred resting habitats and ultimately negative population level effects. These results have implications for new proposed legislation aiming to reduce dolphin exposure to human activities.

## 1. Introduction

The habitat selection strategies of free-ranging animals are driven by trade-offs between the availability of resources necessary for survival, such as prey [1], shelter [2] and the risk of predation [1]. The costs and benefits associated with selecting one habitat over another shape the evolution of behavioural strategies which, in turn, influences individual fitness [3,4]. Habitats that provide optimal combinations of resources are important for population viability [3]. Concerns can arise, however, when the use of important habitats by wildlife overlaps with the repeated use of the same habitats by humans [5,6].

The effect of repeated exposure to human activities on wildlife populations is a growing concern for conservation management. Repeated interactions with humans can lead to changes in many aspects of animal behaviour, influencing activity budgets [7], energetics [8,9], vigilance [10], physiological stress [11,12], reproductive success [13,14], social interactions with conspecifics [15], behavioural patterns [16] and habitat use [17]. These effects can have negative impacts on individual vital rates [18,19], resulting in negative consequences for population viability [20,21].

Since the early 1980s, human interactions with free-ranging cetaceans has increased dramatically worldwide [22,23]. Many coastal cetacean populations are now exposed to prolonged and close-up human encounters causing disruptions to natural behavioural patterns [7,16,24–26]. In the short term, cetaceans are able to compensate for temporary behavioural disruptions [27] by, for example, feeding at other times and/or at other locations [1]. The effects of a short-term behavioural response to disturbance are likely to have little long-term consequence on individual animals [28]. However, the cumulative effect of repeated short-term disturbances may lead to long-term biologically significant consequences. For example, an individual may decide that a higher level of vigilance in a preferred habitat, in which they are repeatedly disturbed, is less costly than moving to an alternative undisturbed habitat that exposes them to greater predation risk. These decisions, however, may be based on the lack of alternative choices, e.g. absence of suitable alternative habitats [17,29] and the condition or capabilities of individuals, e.g. animals may be too weak to relocate [30]. As a consequence, individuals may have no option but to remain and endure repeated disturbance [17,29,30], which could ultimately lead to negative effects on vital rates (e.g. survival and reproduction).

Spinner dolphins (*Stenella longirostris*) in Hawaii exist in small [31,32], genetically isolated populations with restricted ranges [33] and have evolved a constrained diel behavioural pattern. They cooperatively forage offshore at night and return to sheltered bays to socialize and rest during the day [6,34–36]. This spatial and temporal partitioning of behaviours allows the spinner dolphins to maximize their foraging efficiency, while avoiding predation during periods of recovery [10,37].

During periods of activity, animals usually exhibit enhanced brain function, which is often referred to as vigilance [38]. Vigilance is required for many activities including foraging, socializing and predator avoidance. As animals undertake these cognitively challenging activities they tire and accrue what is referred to as a vigilance decrement [38]. Vigilance decrements can manifest as a decreased ability to detect predators or prey [38]. To recover from their energetic and cognitively challenging night-time foraging activities [36,39], spinner dolphins need to rest [40]. Resting in bottlenose dolphins (*Tursiops* spp.), a species that also inhabits coastal areas subjected to strong human influence, has been highlighted as the most sensitive activity to interactions with humans [41]. This could be even more so for spinner dolphins, given the constrained nature of their daily behavioural schedule [10,42].

Spinner dolphins in Hawaii are targeted on a daily basis by humans for close-up encounters [5]. In the waters off the Kona coast, on the leeward side of Hawaii Island spinner dolphins are often observed within four bays during the day: Makako Bay, Kealakekua Bay, Honaunau Bay and Kauhako Bay. Throughout the day, spinner dolphins are repeatedly approached by kayakers, swimmers and vessels inside and outside their preferred resting habitats [6]. Concerns have been raised regarding the effects of the repeated interruption of spinner dolphin resting occasions and have prompted the United States National Oceanic and Atmospheric Administration (NOAA) to look at developing management strategies that reduce the number and intensity of human–dolphin interactions in Hawaii [43].

We collected and analysed data to determine the daytime cumulative activity budget and exposure of spinner dolphins to human activities, both inside and outside their preferred resting habitats, and investigated the effects human activities might be having on the dolphins' daytime cumulative activity budget. Our aim was to inform NOAA to assist in the development of effective human–dolphin management strategies.

## 2. Material and methods

**Figure 1.**
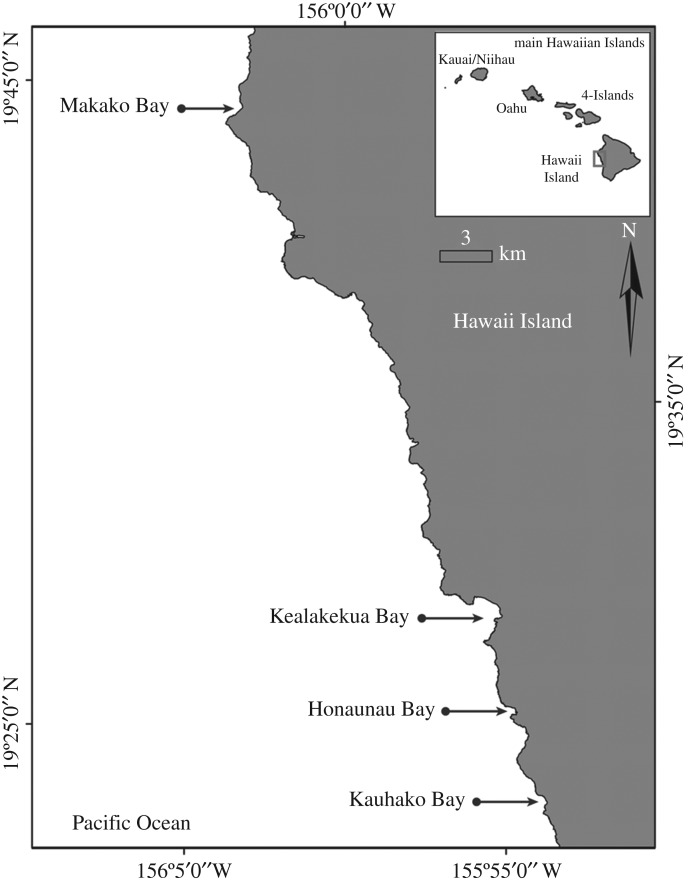
Map of the study area illustrating the four spinner dolphin resting bays, Makako Bay, Kealakekua Bay, Honaunau Bay and Kauhako Bay, along the Kona Coast of Hawaii Island.

To estimate the daytime cumulative activity budget of the spinner dolphins, their exposure to human activities and to investigate the effects of the human activities on the spinner dolphin daytime cumulative activity budget, we used data from four different sampling methods collected inside and outside four spinner dolphin resting bays along the Kona Coast of Hawaii Island (figure 1). Photographic identification to provide individual dolphin identification and the number of observations of each dolphin in each bay, passive acoustic monitoring in each bay to provide daytime presence/absence of dolphins inside the bays and land-based and boat-based group focal follows to collect time-series behavioural data and human activity data inside and outside the four bays.

### 2.1. Data collection

#### 2.1.1. Systematic photo-identification

Between September 2010 and December 2012, boat-based photographic-identification surveys of the Hawaii Island spinner dolphins were carried out in four preferred resting bays, Makako Bay, Kealakekua Bay, Honaunau Bay and Kauhako Bay (figure 1) following a systematic sampling design developed by Tyne *et al*. [31]. To provide consistent effort throughout the study period, each bay was surveyed on the same dates each month: 4 days in Kauhako Bay; 2 days in Honaunau Bay; 4 days in Kealakekua Bay and 2 days in Makako Bay (see [31] for protocol). These data provided individual spinner dolphin identification and the number of times individual dolphins were observed in each bay.

#### 2.1.2. Passive acoustic recordings

To provide the presence/absence data of spinner dolphins within each of the four resting bays, on each day from January 2011 to August 2012, calibrated acoustic recordings of 30 s duration were made every 4 min at a sampling rate of 80 kHz via bottom-mounted DSG-Ocean acoustic instruments (Loggerhead Instruments, Sarasota, FL, USA). Recorders were equipped with HTI-96-Min/3 V hydrophones (Frequency range: 0 Hz–40 kHz) (sensitivity: within 1 dB of −186.6 dbV µPa^−1^, High Tech Inc, Gulfport, MS, USA), a 16-bit computer board and 30.9 GB SD cards. Acoustic data were retrieved approximately every two weeks [44].

Daily daytime spectrograms were generated primarily in Raven Pro 1.5 (Cornell University) and XBAT (Cornell University), a bioacoustics toolbox in Matlab. Spectrograms were generated using a 512-point DFT, 50% overlap and a 512-point (6.4 ms) Hann window. The presence of dolphin sounds was investigated for each bay during daytime through manual visual inspection of the daily spectrogram and was used to document the presence/absence of dolphins based on whether sounds had been documented on recordings. Dolphin sounds included whistles, burst pulse sounds and echolocation. In all cases, we viewed a window of 12 s at a time. If we found dolphin sounds, visual inspection stopped at that time, the time of ‘first dolphin sound’ was noted and the observed day was marked as ‘dolphins present’. To avoid misidentification of background noise, we used echolocation as an indicator of dolphin presence if the echolocation was clear and unambiguous or followed by other dolphin sounds. Days with interrupted recordings (i.e. acoustic logger servicing) were excluded from the analysis of the time of first dolphin sound and days with malfunctions were completely excluded from this analysis (for more details, see [44]).

#### 2.1.3. Group focal follows

Established group focal follow protocols were employed to collect positional, behavioural and human activity information on spinner dolphins during daylight hours from both boat-based and land-based (theodolite observations) platforms. Boat-based dolphin group focal follows were undertaken both inside and outside (within 1 km of the coastline) of these bays to record spinner dolphin time-series group behaviour (see [6] for protocol). Land-based group focal follows were undertaken via theodolite tracking from clifftops overlooking Kauhako Bay (50 m elevation) and Kealakekua Bay (140 m elevation). Group focal follows consisted of a combination of continuous and instantaneous scan sampling procedures [45,46]. Instantaneous scan sampling recorded the predominant group activity, resting, socializing and travelling (table 1) at 10 min intervals [24,45,46]. Behavioural data were categorized as control (undisturbed behaviour) or impact situations. A control situation was when no kayaks, swimmers or boats were with 100 m of the focal group, while an impact situation was when a kayak, swimmer and/or boat was within 100 m of the focal group. A group focal follow was terminated when dolphin behaviour could no longer be reliably determined because of poor visibility, dolphins moving out of range or splitting into too many groups. Time-series data of group behaviour were used to estimate spinner dolphin daytime cumulative activity budgets, to determine the exposure of dolphins to human activities within 100 m and to investigate whether the human activities influenced the spinner dolphin daytime cumulative activity budget. To minimize the impact of the presence of the research vessel on the spinner dolphins during boat-based group focal follows, the vessel was maintained at a distance of 100 m from the focal group and was positioned behind and to the side of the group. All care was taken to minimize disturbance and changes in the dolphin group behaviour induced by the presence of the vessel. Further details of boat-based and land-based group focal follow protocols are given in Tyne *et al*. [6].

**Table 1.** Definitions of spinner dolphin group activities, adapted from Norris *et al*. [35].

predominant group activity	
rest	characterized by tight group, slow speed moving back forth or meandering movement. Individuals typically take multiple breaths; synchronous group diving; changing direction while underwater; spend long periods of time submerged (1.5–3 min); reduced acoustic activity
social	characterized by regular, consistent, aerial behaviours within the group; little time is spent below the surface; dives are brief
travel	characterized by regular and consistent spatial progress with respect to the bottom (in practice surface and shoreline features), i.e. directed swimming that is roughly straight. Travel speed is typically 3.2 km h^−1^

### 2.2. Data modelling and analysis

#### 2.2.1. Calculation of daytime dolphin activity budgets for each bay

Daytime activity budgets are defined as the proportion of time dolphins spent in each activity state (table 1), in each bay and outside the bays and were estimated from the boat-based and land-based group focal follow behavioural time-series data. Boat-based data were used to estimate dolphin activity budgets for Makako Bay, Honaunau Bay and outside the bays. Boat-based and land-based data were combined to estimate dolphin activity budgets within Kauhako Bay and Kealakekua Bay. Activity states were drawn at random (with replacement) from the original dataset (resting, socializing and travelling). This was repeated 1000 times to obtain a density distribution of the relative proportion of time the dolphins spent in each activity state (resting, socializing and travelling) in the activity budget for each bay and outside the bays.

#### 2.2.2. Daytime cumulative activity budgets of individual dolphins

**Figure 2.**
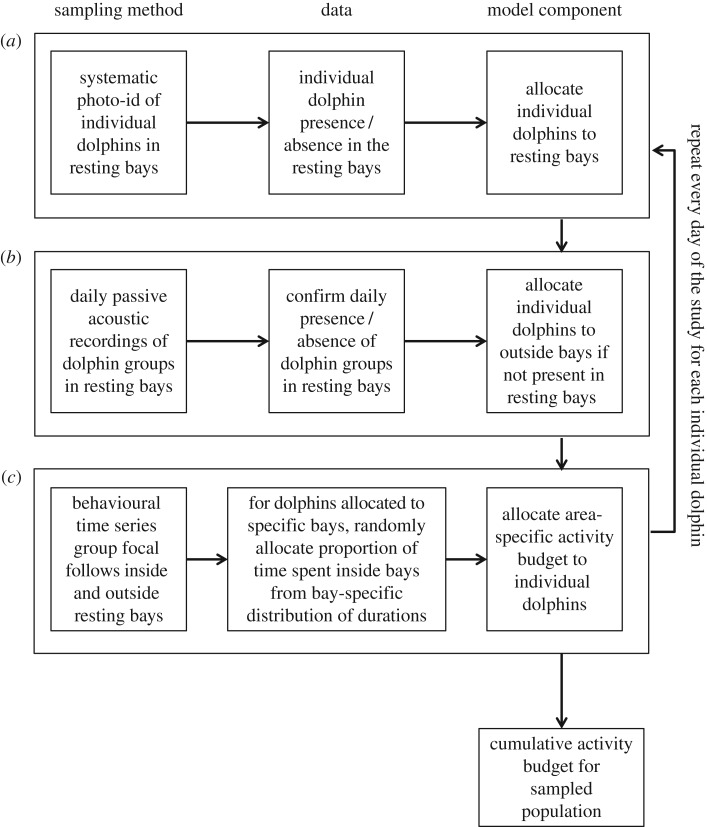
Integration of three discrete datasets to model the cumulative activity budget of spinner dolphins inside and outside of resting bays along the Kona Coast of Hawaii Island. (*a*) Individual spinner dolphin presence/absence in resting bays from systematic photo-id sampling, (*b*) dolphin group presence/absence in resting bays from 24 h per day of acoustic monitoring in resting bays and (*c*) dolphin group behavioural time series from group focal follows inside and outside of resting bays.

The daytime cumulative activity budget represents the daytime (6.00–18.00) activity budget (resting, socializing and travelling) of individual dolphins throughout the study period and takes into consideration the relative time that individual dolphins spent inside and outside of each of the four bays (figure 2). It also considers variations in dolphin activity between different bays and outside the bays (figure 2) and was used to evaluate the effects of human activities on the daytime cumulative activity budget.

Model simulations were used to estimate the daytime cumulative activity budgets of individual photographically identified dolphins (*n* = 235). For each day of the study period, the model randomly allocated individual dolphins to different bays based on their relative occurrence in the bays. The relative occurrence of individual dolphins in each bay was based on the number of times each individual was observed in each bay provided by the photo-identification data (figure 2*a*). After allocating dolphins to bays, the passive acoustic data were used to confirm if dolphins had indeed visited a bay on a given day (binary response based on whether dolphin sounds had been documented in recordings made within each bay on each given day). If a bay had not been visited by dolphins on a given day, the dolphins allocated to that bay were removed and allocated to outside the bays (figure 2*b*). For dolphins that had been allocated to a specific bay, the proportion of time spent inside the bay was randomly drawn from a bay-specific distribution of times dolphins had spent inside the bay (estimated from the focal follow data). If no acoustic data were available for a given day (e.g. because of equipment malfunctions), dolphin presence was drawn at random for that day using a Bernoulli process informed by the probability that dolphins would be present in that bay (dolphin presence/number of observations). Based on the bay which an individual dolphin had been allocated, and the duration of time it spent there, the daily activity budget of the dolphin was calculated (figure 2*c*):

$$\text{Daily activity budget}=(a_{i}\times \text{budget inside ba}y_{i})+(b\times \text{budget outside bays}),$$

where *a* is the proportion of time spent inside bay *i* on a specific day (drawn from a random distribution for bay *i*) and *b* is the proportion of time spent outside of bays (*b* = 1 − *a_i_*). To account for uncertainty in the bay-specific activity budget, a random proportion of the activity budget was drawn from the density distributions that were obtained for the specific bay (see section Calculation of daytime dolphin activity budgets for each bay) and allocated to a dolphin. The same was done for the activity budget outside of bays. Hence, the daily activity budget represents the proportion of time an individual dolphin spent resting, socializing and travelling on a specific day, based on the bay-specific activity budget and the proportion of time spent within a specific bay. Dolphins were assumed to only visit a single bay during a day.

The above procedure was repeated for every day of the study period (*n* = 601 days). The cumulative activity budget was then estimated for every individual dolphin by taking the sum of the duration of the different activity states throughout the study period (figure 2).

All calculations were performed using R 3.0 [47].

#### 2.2.3. Factors affecting daytime dolphin activity states

To better understand which factors influence spinner dolphin daytime activity states, boat-based and land-based observational data were used to determine how the probability of dolphin resting, socializing and travelling was affected by different covariates, including: time-of-day (hour); day-of-year; location (inside or outside bays); number of boats/kayaks/swimmers present (within 100 m of the dolphin group) and distance between dolphin group and boats/kayaks/swimmers. Generalized additive mixed models (GAMM; gamm in R package mgcv) were used with a thin plate regression spline smoother and a binomial distribution and logit link function. In the model selection process, covariates and interactions between covariates were added sequentially to the null model. The *F*-statistic for the ANOVA *F*-test was estimated for each model and compared with that of the previous model. Covariates were added both as linear effects and nonlinear smoothers to cover all possible relationships between the response and explanatory variables. As sequential observations within focal follows could not be considered independent, a temporal auto-correlation structure within follows was incorporated in the model, where the residuals at any given time were modelled as a function of the residuals of the previous time points. The most suitable auto-correlation structure was fitted by altering the number of auto-regressive and moving average parameters and then comparing the different models. Auto-correlation and partial auto-correlation function plots were used to detect patterns of auto-regressive and moving average parameters visually, before and after adding the different correlation structures.

The variance inflation factor (VIF) was used to investigate collinearity (high correlation) between the explanatory variables in the model. A threshold value of three was used to remove collinear variables one at a time until all VIF values were below three and no collinearity remained [48]. For all models, model validation tests were run to identify potential violations of assumptions. Scatter plots of residuals versus fitted values and residuals against each explanatory variable were used to test the assumption of equal variances (i.e. homogeneity of variance) in the model. Normality of residuals was interpreted from quantile–quantile plots and from residual histograms. Over-dispersion was tested for each model by dividing the residual deviance by the residual degrees of freedom and a value of greater than 1.5 was used to indicate over-dispersion [49].

#### 2.2.4. Frequency of human interactions

To investigate the elapsed time between human interactions with dolphins, frequency histograms of *unapproached* (continuous time with no human interactions with dolphins) and *approached* (continuous time with human interactions with dolphins) durations were inspected and compared.

## 3. Results

### 3.1. Summary of photo-identification and behavioural sampling efforts

**Figure 3.**
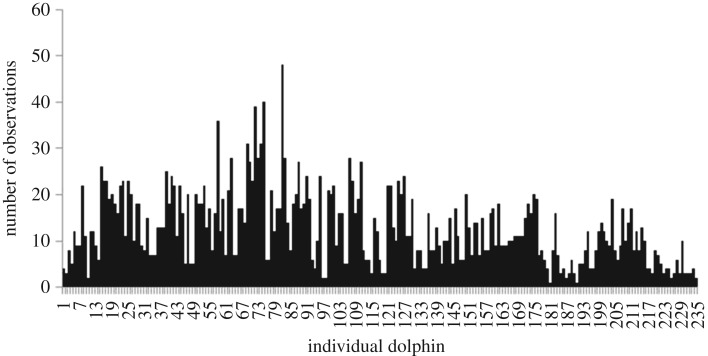
The number of sightings of each of the 235 photographically identified spinner dolphins in Makako Bay, Kealakekua Bay, Honaunau Bay and Kauhako Bay between September 2010 and August 2012.

The systematic sampling design developed in Tyne *et al*. [31] to collect individual spinner dolphin photo-identification data resulted in nearly 2500 h of on-water effort over 276 days of sampling (table 2). From these data, 235 highly distinctive individual spinner dolphins were identified. The number of individual dolphin sightings ranged from 1 to 48 during the study period (figure 3), with a mean of 12 ± 0.52 (±1 s.e.).

**Table 2.** Number of days, hours and mean length of photo-identification surveys conducted within the four resting bays between September 2010 and August 2012.

location	survey days	hours	mean survey length (hr:min ± s.e.)
Makako Bay	46	395	8:35 ± 0:11
Kealakekua Bay	92	818	8:53 ± 0:13
Honaunau Bay	46	412	8:57 ± 0:15
Kauhako Bay	92	856	9:18 ± 0:10
total	276	2481	9:00 ± 0.12

A total of 105 boat- and land-based dolphin group focal follows was conducted over approximately 428 h, of which 75 (71.4%) were from the boat-based platform and 30 (28.6%) were from the land-based platform (table 3).

**Table 3.** Number and duration of dolphin group focal follows collected from land-based and boat-based platforms inside bays and outside of resting bays along the Kona Coast, Hawaii Island.

focal follow	number of focal follows	total focal follow hours	mean focal follow duration (hr:min ± s.e.)
land-based			
Kealakekua Bay	23	189	8:27 ± 0:19
Kauhako Bay	7	38	3:25 ± 1:17
total	30	227	7:57 ± 0:22
boat-based			
Makako Bay	13	26	2:00 ± 0:26
Kealakekua Bay	10	16	1:36 ± 0:15
Honaunau Bay	5	21	4:12 ± 0:15
Kauhako Bay	10	16	1:36 ± 0:48
total	38	117	2:21 ± 0:26
outside bays	37	84	3:10 ± 0:13
total	75	201	2:41 ± 0:10
overall total	105	428	4:08 ± 0:51

### 3.2. Passive acoustic monitoring efforts

In each of the four bays, bottom-mounted acoustic loggers were simultaneously deployed for 601 days. A total of 2148 recording days were made over the study period: 565 days in Makako Bay, 484 days in Kealakekua Bay, 563 days in Honaunau Bay and 536 days in Kauhako Bay [44]. Acoustic recordings confirmed the daytime presence of dolphins during 90% of monitoring days in Makako Bay, 65% in Kealakekua Bay, 37% in Honaunau Bay and 51% in Kauhako Bay (for more details see [44]).

### 3.3. Daytime bay use by individual dolphins

**Figure 4.**
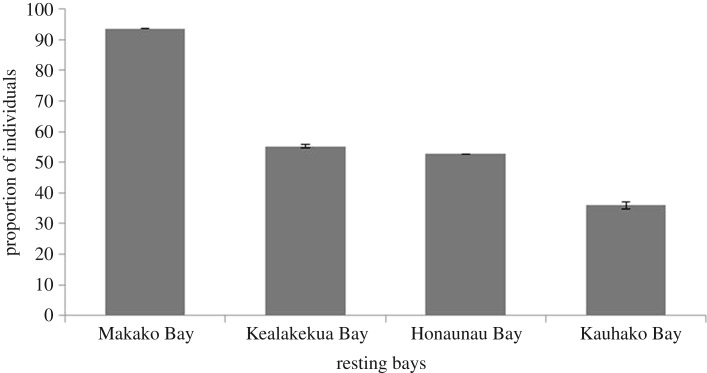
The proportion of the 235 identified spinner dolphins that were documented in each of the four resting bays. Data from Kealakekua Bay and Kauhako Bay were standardized and 95% confidence interval error bars are shown.

Most dolphins (94%, *n* = 220) were observed in Makako Bay, followed by Kealakekua Bay (55%, *n* = 130), Honaunau Bay (53%, *n* = 124) and Kauhako Bay (36%, *n* = 85; figure 4). Some dolphins showed a strong preference for a specific bay, while others showed less preference and could be observed in two or more bays throughout the study period (electronic supplementary material, figure S1).

### 3.4. Daytime bay-specific activity budgets

**Figure 5.**
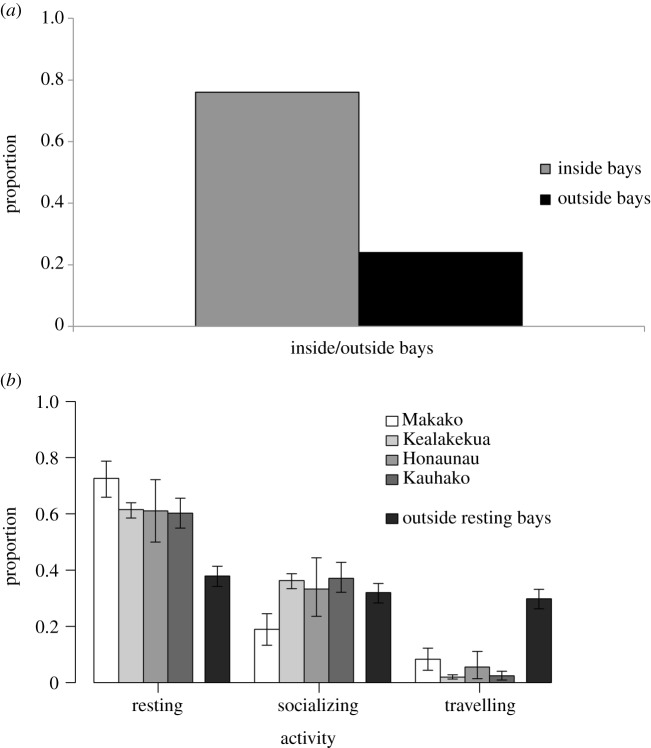
(*a*) The proportion of daytime spinner dolphins spent inside and outside of sheltered bays and; (*b*) the proportion of time dolphins were in resting, socializing and travelling activity states while inside and outside of the four resting bays along the Kona Coast, Hawaii Island. Error bars represent 95% highest posterior density intervals, analogous to 95% confidence intervals.

Model simulations estimated that during the daytime individual spinner dolphins spent most of their time inside bays (76% of time), while spending 24% of the time outside bays (figure 5*a*). Spinner dolphins spent most of the daytime resting, followed by socializing and travelling (figure 5*b*). The proportion of daytime spent resting was higher inside bays (greater than 60%) than outside (less than 40%; figure 5*b*), but as the dolphins only spent 24% of the daytime outside bays, overall the proportion of time they rested outside bays was less than 10%. Time spent socializing was approximately the same (approx. 35%) inside and outside bays, dolphins spent a substantially higher proportion of time travelling outside (approx. 30%) than inside bays (approx. 5%; figure 5*b*). There was little variation in the dolphins' activity budget between bays, with the exception of Makako Bay, where dolphins spent more time resting (72.6%), and less time socializing (19.0%) than in the other bays (figure 5*b*).

### 3.5. Daytime cumulative activity budget

The daytime cumulative activity budget showed that individual dolphins spent between 49.5% and 69.4% of the daytime resting (mean = 61.7%, s.d. = 6.5). Of the time spinner dolphins spent in Makako Bay, the dolphins spent a higher proportion of time resting than in the other bays (figure 5*b*). Socializing activity showed a similar pattern, with dolphins spending between 20.2 and 34.7% of their daytime socializing (mean = 26.1%, s.d. = 5.0). While both resting and socializing activities showed large individual variations, there were small variations in the proportion of daytime dolphins spent travelling, ranging between 10.3 and 16.9% of their time (mean = 12.2%, s.d. = 1.6).

### 3.6. Factors affecting dolphin daytime activity

None of the human activity covariates (presence of boats/kayaks/swimmers (*p* = 0.995), distance between dolphins and boats/kayaks/swimmers) had a significant effect on the probability of dolphins resting, socializing or travelling. All human activity covariates (presence of boat/kayak/swimmers within 100 m) were collinear, preventing the use of more than one covariate in each model. The control data (no boats/kayaks/swimmers present within 100 m) constituted only 27.7% of the land-based data and 5.3% of the boat-based data. In total, spinner dolphins were exposed to human activities (impact scenario; human activity within 100 m) during 82.7% of the time (focal follow observations).

### 3.7. Frequency of human interactions

**Figure 6.**
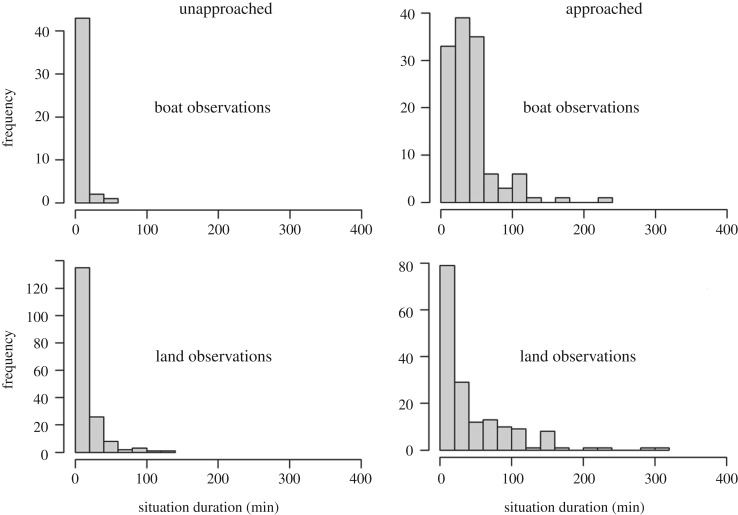
Frequency histograms depicting the duration (min) of time periods when spinner dolphins were observed in ‘approached’ or ‘unapproached’ situations, i.e. continuous time spent in approached or unapproached situations. Data are based on focal follow data collected from boat-based (top row) and land-based (bottom row) platforms.

The proportion of time that dolphins were exposed to boats, kayaks and swimmers differed between bays (electronic supplementary material, figure S2). Inside Makako Bay, dolphins were predominately exposed to boats and swimmers, but not to kayaks (electronic supplementary material, figure S2). By contrast, in the other bays, dolphins were mostly exposed to kayaks and swimmers (electronic supplementary material, figure S2). Frequency histograms depicting the duration of each time period when spinner dolphins were observed in ‘approached’ or ‘unapproached’ situations (i.e. continuous time spent in each situation) highlight that unapproached situations were significantly shorter compared with approached situations (figure 6). The median unapproached situation durations were 10 min for both the boat- and land-based data, while the corresponding approached durations were 70 and 30 min, respectively.

## 4. Discussion

During daytime hours, spinner dolphins were exposed to human activities within 100 m for greater than 82% of the time. To our knowledge, this level of exposure is substantially greater than those previously reported for any other dolphin species (table 4). Despite the high level of exposure, however, human activities seemingly did not have a significant effect on the probability of spinner dolphins resting. This result is, however, probably an artefact of the low level of control data available (less than 18% of observations) to make robust comparisons between behavioural patterns in unapproached (continuous time periods with no human activity within 100 m of dolphins) and approached situations (continuous time periods with human activities within 100 m). Furthermore, the elapsed time between repeated disturbances can also influence the activity budget of dolphins [16], as exposed animals generally need time to return to their initial activity state following an interaction [24,51]. Insufficient time between interactions may prevent dolphins from returning to their initial activity state. During approached situations, spinner dolphins were exposed to human activities for between 30 and 70 min. Unapproached situations had a median duration of 10 min before dolphins were exposed to human activity again. Dolphins need time to recover from a disturbance to return to a pre-disturbed activity state [16,24]. For example, bottlenose dolphins in Milford Sound, New Zealand, required at least 68 min between interactions to return to their pre-disturbed behaviour [16]. It is likely that the short time intervals between successive exposure events are insufficient for spinner dolphins to return to a natural behavioural state between exposures. Consequently, the unapproached observations, as defined in this current study, may not accurately represent the natural resting behaviour of spinner dolphins, but may represent one of a more vigilant nature, reflecting that the dolphins may not be achieving a natural resting state. This may also explain why no significant differences were detected in the probabilities of observing resting activity states during approached and unapproached situations.

**Table 4.** Studies that have quantified exposure rates of dolphins to human activities and whether authors noted or inferred an impact. MV, motorized vessels; K, Kayaks; SUP, stand-up paddleboard; S, swimmers.

species	proportion of time exposed to human activities %	impact distance (m)	source of disturbance	behavioural response	study
Bottlenose dolphin (*Tursiops truncatus*)	9	400	MV, K	yes	[7]
Bottlenose dolphin (*T. truncatus*)	10.8	400	MV, K	yes	[16]
Bottlenose dolphin (*T. truncatus*)	12.8	400	MV, K	yes	[16]
Bottlenose dolphin (*T. truncatus*)	15.5	400	MV, K	yes	[50]
Common dolphin (*Delphinus* sp.)	21	300	MV, SUP, K	yes	[51]
Hector's dolphin (*Hectori hectori*)	23.6	200	MV	no	[52]
Bottlenose dolphin *T. truncatus*	24	50	MV, S	yes	[53]
Common dolphin (*Delphinus* sp.)	29	300	MV	yes	[54]
Killer whale (*Orcinus orca*)	28.5	100	MV	yes	[55]
Dusky dolphin (*Lagenorhynchus obscurus*)	31	200	MV	yes	[56]
Killer whale (*O. orca*)	37.6	100	MV	yes	[55]
Bottlenose dolphin (*T. truncatus*)	45	50	MV, S	yes	[57]
Dusky dolphin (*L. obscurus)*	51.6	300	MV	yes	[58]
Bottlenose dolphin (*T. truncatus*)	58	300	MV	yes	[26]
Spinner dolphin (*S. longirostris*)	77	300	MV, K, S	not reported	[59]
Spinner dolphin (*S. longirostris*)	26, 42 and 53^a^	300	MV, S	yes	[60]
Spinner dolphin (*S. longirostris*)	82.7	100	MV, K, S	n.d.	this study

^a^The proportion of time the spinner dolphins were exposed to human activities in three different areas in the Red Sea, Egypt.

Spinner dolphins spent most of the daytime hours (76%) inside sheltered bays and 24% of the daytime outside of these bays. Of the daytime spinner dolphins spent outside the bays, nearly 40% of that time was spent resting; this indicates that the spinner dolphins are less likely to rest (less than 10% of the daytime) outside of the bays [6]. However, should dolphins increase the time they spend outside the bays and the proportion of time they rest outside the bays, it may suggest that the dolphins are being displaced from their preferred resting areas and resting in less suitable habitats, which may lead to an increased predation risk.

Spinner dolphins spent the highest proportion of their time resting in Makako Bay compared with the other bays, and most (94%) of the identified dolphins were observed in the bay even though boats and swimmers are the main human activities. Previous research suggests that resting spinner dolphins are acoustically silent [35]. By contrast, however, recent results suggest that resting spinner dolphins can be acoustically active in the presence of human activities in Makako Bay [61]. This could be an indication that resting behaviour in Makako Bay is one of a more vigilant nature. Makako Bay is also proximal to spinner dolphin night-time foraging areas [37], which is important in the selection of spinner dolphin resting habitat [37]. The high proportion of resting time in Makako Bay could be a trade-off between vigilant rest in a preferred habitat with a high proportion of human activity, rather than deeper rest in a less suitable habitat, with less human activity but where the dolphins may be more vulnerable to predation.

The constrained diel behavioural schedule of spinner dolphins [42] may affect their ability to compensate if deprived of rest [42], and they may not sufficiently recover from their night-time foraging-induced vigilance decrement [38], leading to impaired cognitive and decision-making abilities [62–64]; in turn, potentially reducing their ability to detect predators, a reduced foraging efficiency, reduced reproductive success and compromised social skills. During night-time foraging bouts, spinner dolphins cooperatively herd prey into dense aggregations and pairs of spinner dolphins then take turns to forage within these aggregations [36]. Impaired cognition, may affect the success of this cooperative foraging strategy [36], by compromising the development and reinforcement of social bonds between conspecifics or to properly perceive prey patches during foraging activities. Impaired cognition can adversely affect the social cohesion of a community [15]. Moreover, mothers and calves may be particularly susceptible to rest deprivation if the ability of mothers to properly care for, feed and protect their calves is compromised [14,65].

Elsewhere, dolphin communities with considerably less cumulative exposure to human activities (table 4) have had their natural behavioural patterns disrupted [24–26] and energy budgets affected [8]. Repeated exposure to human activities has also resulted in long-term habitat abandonment [16,24], which has led to longer-term strategies such as the avoidance of important habitats [16], and subsequently biologically negative impacts on populations [18,66,67]. For example, in Doubtful Sound, New Zealand, the short-term avoidance of tour boats by bottlenose dolphins led to a long-term avoidance of preferred habitat [16,66] and in Shark Bay, Western Australia, the relative abundance of bottlenose dolphins within a tourism area declined in response to an increase in tour vessel activity [67]. It is likely that the spinner dolphins of Hawaii may be under similar pressure. In fact, signs of displacement are emerging [42]. Over the past three decades, human activities in spinner dolphin resting bays have increased significantly [68]. Concurrently, the most recent spinner dolphin abundance estimates 631 (95% CI 524–761) [31] and 668 (95% CI 556–801) [32] are lower than all previous estimates 960 [35], 2334 [69], 1001 and 855 [70], indicating a possible long-term impact. Furthermore, the anthropogenic underwater sound was not considered during this study, but clearly affects the soundscape of the four bays and animals that rely on acoustics as their main sensory input [71]. As such, our estimates of spinner dolphin exposure to human activities is conservative as it does not account for engine noise which can propagate many kilometres [72], further highlighting the need for management actions that aim to reduce the exposure of spinner dolphins to human activities within these important resting habitats.

## Data accessibility

The datasets supporting this article are available from the Dryad data repository: http://dx.doi.org/10.5061/dryad.332pb [73].
